# *n*-Type Doping of Vapor–Liquid–Solid Grown GaAs Nanowires

**DOI:** 10.1007/s11671-010-9815-7

**Published:** 2010-10-07

**Authors:** Christoph Gutsche, Andrey Lysov, Ingo Regolin, Kai Blekker, Werner Prost, Franz-Josef Tegude

**Affiliations:** 1 Solid State Electronics Department and CeNIDE, University of Duisburg-Essen, Lotharstr. 55, 47048, Duisburg, Germany

**Keywords:** Nanowires, MOVPE, Gallium arsenide, Doping, Silicon, Tin, Optoelectronics

## Abstract

In this letter, *n*-type doping of GaAs nanowires grown by metal–organic vapor phase epitaxy in the vapor–liquid–solid growth mode on (111)B GaAs substrates is reported. A low growth temperature of 400°C is adjusted in order to exclude shell growth. The impact of doping precursors on the morphology of GaAs nanowires was investigated. Tetraethyl tin as doping precursor enables heavily *n*-type doped GaAs nanowires in a relatively small process window while no doping effect could be found for ditertiarybutylsilane. Electrical measurements carried out on single nanowires reveal an axially non-uniform doping profile. Within a number of wires from the same run, the donor concentrations *N*_D_ of GaAs nanowires are found to vary from 7 × 10^17^ cm^-3^ to 2 × 10^18^ cm^-3^. The *n*-type conductivity is proven by the transfer characteristics of fabricated nanowire metal–insulator-semiconductor field-effect transistor devices.

## Introduction

Novel, quasi one-dimensional structures, like III-V semiconductor nanowires, may act as key elements in future nanoscaled optoelectronic devices [1-3]. They offer intriguing electrical and optoelectronic properties and the ability to combine material systems that are impossible in conventional semiconductor layer growth due to lattice mismatch issues [4]. The large surface to volume ratio, which is already utilized in nanowire sensor applications [5,6], allows to improve light extraction and light collections when compared to planar devices making especially nanowires ideal candidates for light emitters and photo voltaics [7-9]. However, the future of any semiconductor nanowire technology will inherently rely on their doping capability. Only this way, the control of carrier type and density representing the unique advantage of semiconductors will be available [3]. Unfortunately, the specific parameters for nanowire growth do often not favor the incorporation of doping atoms. Moreover, both *n*- and *p*-type doping within the same semiconductor has to be provided for most optoelectronic applications.

There are only a very few publications describing initial doping results of III-V compound semiconductor nanowires with a high charge carrier density. Most of them focus on the material systems InAs [10] and InN [11], which is not astounding since at the surface of these semiconductors, the surface Fermi level is pinned [12] in the conduction band. This effect makes *n*-type conductivity easy to the expense of difficulties for *p*-type doping. In other semiconductors like GaAs, the Fermi level at the surface is pinned approximately in the center of the band gap resulting in a substantial surface depletion that may lead to non-conducting nanowires even at elevated doping levels. On the other hand, both a controlled *p*- and *n*-type doping might be available. Doping of GaAs nanowires grown by molecular beam epitaxy (MBE) has been demonstrated in different means. LaPierre et al. used Be and Te as *p*- and *n*-type dopant precursors [13], while Fontcuberta i Morral et al. pointed out that Si may act as both by just changing the operating temperature during growth [14,15]. The incorporation of Si and Be into GaAs nanowires was investigated in a further study [16]. Nevertheless, the growth and dopant mechanisms of GaAs nanowires grown by MBE differ to some extend from chemical vapor deposition (CVD) methods, since the growth temperatures of the first-mentioned are usually much higher (500°C < Tg < 650°C). Till now, just in case of InP nanowires, both a successful *n*- and *p*-type doping, respectively, have been obtained in the core of untapered III-V nanowires synthesized via metal–organic vapor phase epitaxial (MOVPE) growth. Here, hydrogen sulfide (H_2_S)/tetraethyl tin (TESn) and diethyl zinc (DEZn)/dimethyl zinc (DMZn) were used as dopant sources [7,17] in the vapor–liquid solid (VLS) growth mode. *p*-doping of VLS-grown GaAs nanowires was demonstrated supplying DEZn during MOVPE growth [18], but a study on *n*-type doping is pending.

In this letter, *n*-type doping of GaAs nanowires grown by VLS using two different precursor materials, ditertiarybutylsilane (DitBuSi) and tetraethyl tin (TESn), is reported. Structural and morphological changes possibly induced by dopant incorporation were analyzed. Ohmic contacts to single *n*-GaAs nanowires and their electrical measurements are described. The *n*-type conductivity is proven by measuring the transfer characteristics of fabricated GaAs nanowire field-effect transistors. By adopting a transport model [18], the carrier concentrations of GaAs:Sn wires are estimated in the presence of surface depletion.

## Experimental

GaAs nanowires were grown on GaAs (111)B substrates by metal–organic vapor phase (MOVPE) epitaxy in an AIX200 RF system with fully non-gaseous source configuration [19]. Monodisperse as well as polydisperse Au nanoparticles were deposited as growth seeds prior to growth. Monodisperse nanoparticles with a diameter of 150 nm were taken from a colloidal solution. Polydisperse metal seeds for VLS growth of the nanowires were formed by evaporation and subsequent annealing of a thin Au layer of nominally 2.5 nm thickness. The anneal step was carried out at 600°C for 5 min under group-V overpressure and resulted in nanoparticles with diameters from 30 nm to some 100 nm. Nanowires were grown at a total pressure of 50 mbar, using Trimethylgallium (TMGa) and Tertiarybutylarsine (TBAs) as precursors with a constant V/III ratio of 2.5. The total gas flow of 3.4 l/min was provided by N_2_ as carrier gas, while H_2_ was used for the bubblers. After the growth start, initiated at 450°C for 3 min, the final growth temperature was adjusted to 400°C, to exclude almost completely additional VS growth on the nanowire side facets [20]. *n*-doping effect was investigated by an additional TESn (0.02 ≤ IV/III ≤ 0.16) or DitBuSi (IV/III ≤ 0.52) supply.

Morphological characterization of the nanowires was performed via scanning electron microscopy (LEO 1530). Electrical results were obtained with standard DC-measurements setup. Therefore, the as-grown structures were transferred to special pre-patterned carriers and finally contacted by electron beam lithography (E-Beam) or optical lithography, respectively. The carrier consists of a semi-insulating GaAs substrate that was covered with 300-nm-thick silicon nitride (SiN_x_) for improved isolation. The ohmic contacts were formed by evaporation of Ge (5 nm)/Ni (10 nm)/Ge (25 nm)/Au (400 nm), which is known to be a typical contact system for *n*-GaAs [21]. To improve the contact properties, a rapid thermal annealing was carried out for 30 s or 300 s at 320°C. In addition, metal–insulator-semiconductor field-effect transistor (MISFET) devices were fabricated with about 30 nm SiN_x_ gate dielectric and Ti/Au gate metal [22] to verify the type of conductivity.

## Results and Discussion

### Growth Results

**Figure 1:**
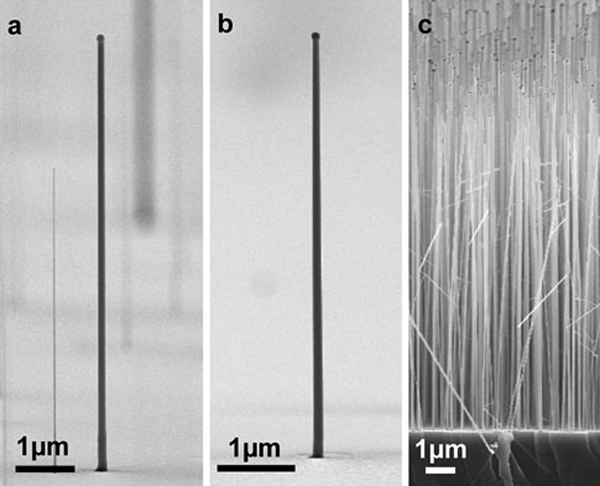
**SEM micrographs of GaAs nanowires grown on GaAs (111)B substrates: *a* from colloidal nanoparticles with 150 nm diameter under TESn supply (IV/III = 0.08), *b* from colloidal nanoparticles with 150 nm diameter under DitBuSi supply (IV/III = 0.52), *c* grown under the same conditions as in a but from polydisperse seed particles formed by annealing of a 2.5 nm Au layer**. The different nanowire density in **a** and **b** is just accidental.

SEM micrographs of three different samples are depicted in Figure 1a–c. The selected growth temperature of 400°C suppresses the conventional layer growth on the side facets [20], leading to a very high aspect ratio up to gr, _VLS_/gr, _VS_ > 1,000. Hence, the doping mechanism through side facet deposition, reported in various publications [14,23], can be excluded. This enables a separate investigation of VLS-grown GaAs nanowires. The wires given in Figure 1a and 1b are grown from colloidal Au seed particles with 150 nm diameter and under supply of TESn (Figure 1a, IV/III = 0.08) and DitBuSi (Figure 1b, IV/III = 0.52), respectively. In addition, nanowires grown from polydisperse seed particles under the same conditions as in (a) are shown in Figure 1c. All of the nanowires adopted the crystal orientation of the growth substrate and are upstanding in (111)B direction. Furthermore, no wire kinking or other structural defects, even at higher TESn supply up to IV/III = 0.16, were observable (for TEM analysis refer to [24]). In contrast, *p*-type doping with diethylzinc (DEZn) revealed a strong influence on the crystal structure, even at low II/III ratios higher than 0.008, as reported previously [18]. One possible reason may be that the solubility of Sn and Si in the Au particle is much lower than for Zn at the selected growth parameters. The phase diagrams of Au–Sn [25], Au–Si [26] and Au-Zn [27] substantiate this assumption, since there exists no eutectic point for the binary Au-Zn alloy at 400°C. Hence, more and more Zn might be solved in the Au particle during the nanowire growth process. With higher II/III ratios, this leads into an increased number of structural defects and wire kinking. For *n*-type doping, using TESn and DitBuSi, respectively, the solubility of dopants in the seed particle is lower, which accounts for the good crystal structure despite relatively high dopant supplies. Of course, the nanoscale may differ to some extent and adding a third component (Gallium) complicates the chemistry/physics at the droplet. Nevertheless, the reported differences regarding *n*- and *p*-type doping become more comprehensible.

### Electrical Characterization

**Figure 2:**
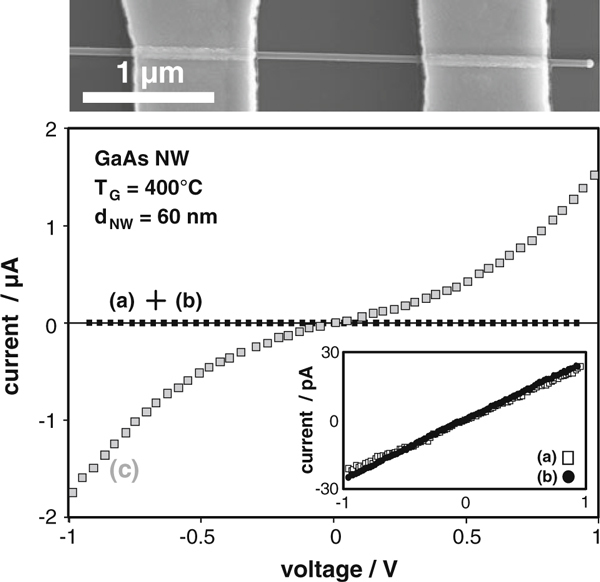
***Top*: SEM image of a GaAs nanowire from sample *c* connected to two electrodes for electrical measurements**. The contact spacing is 1.3 μm. *Bottom*: I-V characteristics of the untapered GaAs nanowires grown at 400°C: *a* grown without dopant supply, *b* grown under supply of DitBuSi (IV/III = 0.52), *c* grown under supply of TESn (IV/III = 0.08). The second *inset* shows the I–V curves of *a* and *b* in a more adequate current scale.

Representative I–V characteristics for nanowires grown without dopant supply, with supply of DitBuSi (IV/III = 0.52) and with supply of TESn (IV/III = 0.08) are displayed in Figure 2. The non-intentional doped (nid) GaAs nanowires let pass a current of a few pA at 1 V applied bias, corresponding to a resistance in the GΩ range. Adding DitBuSi to the gas phase during growth has no remarkable effect on the conductivity of nanowires, even at relatively high IV/III ratios. This can easily be interpreted since Si is an amphoteric impurity in GaAs [28,29]. First, principle calculations claim that this also holds for nanowires [30]. In addition, the growth temperature of 400°C might be to low for a sufficient cracking of the DitBuSi precursor [31]. The latter argument can not be the only reason for the non-existing doping effect using DitBuSi, since we already carried out doping experiments on GaAs nanowire shells at growth temperatures up to 650°C (e.g. same temperature as for GaAs layer growth), which also failed.

If TESn at IV/III = 0.08 is used as dopant precursor, the current of 2 μA at 1 V applied bias is about six orders of magnitude higher than for the nid sample, giving evidence of the doping effect. The corresponding I–V characteristic is not perfectly ohmic, which indicates a small remaining contact barrier, while no blocking region is observable. The realization of ohmic contacts on *n*-GaAs is known to be challenging specially at low annealing temperatures due to the already mentioned Fermi level pinning and high density of surface states [12]. This well-known classical problem becomes much more serious in nanowire devices due to the increase in surface to volume ratio, which in turn complicates the ohmic contact fabrication even on relatively high-doped *n*-GaAs nanowires. However, annealing at higher temperatures than 320°C leads to an increased out-diffusion of Ga into the Au contact layer. This effect is also reported for bulk material [32], but gets crucial in the nanoscale since it destroys the nanowire and has to be avoided. Regarding the following analysis of the doping concentration, it should be noted that the nanowire resistances are extracted for voltages ≥ 1 V, where the remaining contact barrier is just a small series resistance. Therefore, the later given *N*_D_ values might be slightly underestimated, but in the same order of magnitude. Further, we assume that in case of the nid- and Si-doped nanowires, the I–V behavior is dominated by the high wire resistance and hence completely ohmic in the investigated regime.

In order to determine the carrier concentration of the Sn-doped GaAs nanowires, we adopted the model used for *p*-GaAs (for detailed informations see [18]) and exchanged the varying parameters. For (100) *n*-GaAs, the value for the surface potential φ_S_ is 0.6 eV [33]. The dependence between carrier concentration and mobility μ is given by the Hilsum formula [34]:

(1) $$\mu =\mu_{0}/(1+\sqrt{N_{\text{D}}/10^{17}{\text{cm}}^{-3}})$$

Here, we used a value of μ_0_ = 8,000 cm^2^/Vs. It should be pointed out that this is a simplification since the Hilsum formula is employed for bulk material and the carrier mobility μ_0_ is also set to that of bulk GaAs. Therefore, scattering via surface states and stacking faults are not considered. In literature, carrier mobility measured via the transconductance of the nanowire device, which utilizes simplifications to the same degree, reveals lower mobility than known bulk values. If e.g. μ_0_ is reduced to 4,000 cm^2^/Vs, the doping concentration for a nanowire with *r*_NW_ = 100 nm and *R*_NW_(1 μm) = 2 kΩ changes to 2 × 10^18^ cm^-3^, which also suggests that our N_D_s might be underestimated (1 × 10^18^ cm^-3^ for μ_0_ = 8,000 cm^2^/Vs).

**Figure 3:**
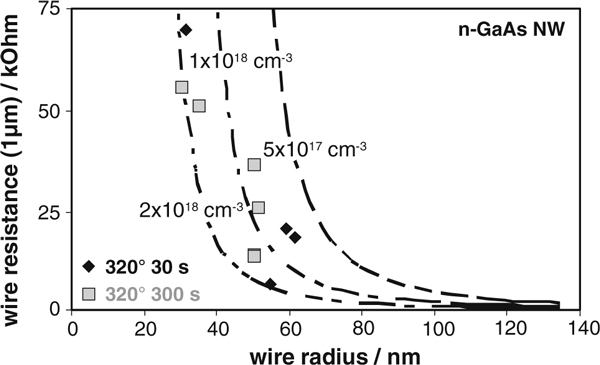
**Measured wire resistance versus the wire radius for a IV/III ratio of 0.08 for two different annealing cycles**. The resistance is normalized to wires with 1-μ length. In addition, modeled data for three different carrier concentrations (5 × 10^17^ cm^-3^, 1 × 10^18^ cm^-3^, 2 × 10^18^ cm^-3^) are given in *dashed lines*.

The electrical conductivity of a number of nanowires with various radii (30 nm < r_0_ < 70 nm) were analyzed in the linear regime. Since the contact resistances were located in the low kOhm range, which is only a few percent of the total device resistance, we neglected it during the following analysis. Taking it into account would again just lead to a marginal shift to slightly higher carrier concentrations. In Figure 3, the corresponding experimental wire resistances for a IV/III ratio of 0.08, normalized to a contact spacing of *L* = 1 μm, are depicted. Rhombuses represent contact annealing for 30 s, rectangles for 300 s, respectively. No dependence on the duration of the annealing step can be observed from this figure. In addition, modeled data for three different values of carrier concentration (5 × 10^17^, 1 × 10^18^, 2 × 10^18^ cm^-3^) are given in dashed lines. The wire resistance decreases with both increasing carrier concentrations and wire radius, respectively. It is evident that the experimental resistance data are spreading between the three modeled lines. We conclude that the doping density *N*_D_ varies in the range of 7 × 10^17^ cm^-3^ ≤ *N*_D_ ≤ 2 × 10^18^ cm^-3^. The spreading is attributed to both a limited precision of geometrical wire data and a possible doping inhomogenity, i.e. a realistic precision of ± 5% in the measurement of the wire diameter and the wire length, respectively, may sum up to a variation of up to ± 15% of the evaluated doping density. The experimental spreading of ± 32% is substantially higher such that an inhomogenity of doping density, which was already reported for GaAs:Zn [18], is assumed.

**Figure 4:**
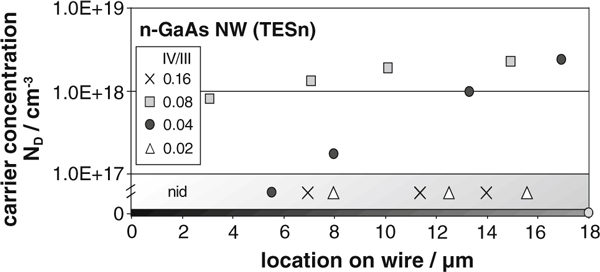
**Carrier concentration against the location on the wire for various IV/III ratios from 0.02 up to 0.16**. Length zero represents the wire bottom. An axially graded doping profile is visible.

In order to investigate whether the doping profile is axially graded, we carried out electrical measurements on different parts of the nanowires separately (e.g. we fabricated four or five contacts along the length of the NW). These measurements were performed on nanowires grown under various IV/III ratios to analyze the correlation between IV/III ratio and carrier concentration additionally. In Figure 4, we plotted the carrier concentration against the location on the wire for IV/III ratios from 0.02 up to 0.16. The given data for the previously described TESn supply (IV/III = 0.08) reveal an axially non-uniform doping profile with *N*_D_ values spreading in the same range as the ones estimated before (7 × 10^17^ cm^-3^ ≤ *N*_D_ ≤ 2 × 10^18^ cm^-3^). We suggest that Sn accumulates within the Au (or Au/Ga, respectively) particle during growth. Hence, the probability of dopant incorporation increases in the same way. Simplified, we conclude that the Au seed particle acts like a first-order time-delay element for the dopant atoms. If the IV/III ratio is decreased (IV/III = 0.04), just the upper part of grown nanowires show heavy doping effect (*N*_D_ ≥ 1 × 10^17^ cm^-3^), with graded carrier concentrations in the same range as described before (see Figure 4 black dots). Recently, Wallentin et al. reported on InP/GaAs esaki diodes, indicating a sharp onset of the doping [35]. We therefore conclude that the lower parts of these nanowires (IV/III = 0.04) are doped at relatively low doping levels (*N*_D_ ≤ 1 × 10^17^ cm^-3^). By further decreasing the dopant supply to a IV/III ratio of 0.02, we observed that the nanowires exhibit the same electrical properties as nid ones over the whole length of about 20 microns. We assume that the amount of dopant atoms accumulated within the Au seed particle during growth is to low to induce a remarkable doping effect. To further increase the carrier concentration (IV/III ratio), we decreased the Ga flow (note that the TESn flow is limited by our mass flow controller configuration), while the As flow was kept constant, leading into an V/III ratio of 5. Hence, we achieved a IV/III ratio of 0.16 that is doubled compared to the standard sample. Curiously, the corresponding I–V characteristics of the contacted nanowires revealed that the conductivity as well as the contact properties was not enhanced, but got even poorer. The current flow was decreased by orders of magnitude, indicating a carrier concentration lower than 1 × 10^17^ cm^-3^ (Figure 4 crosses). In addition, we observed that the growth rate of the nanowires grown at IV/III = 0.16 is higher than for the ones grown at IV/III = 0.08 though the Ga flow is halved (gr_0.16_ ≈ 425 nm/min, gr_0.08_ ≈ 390 nm/min). This effect might be attributed to a higher diffusion length of Ga atoms induced by the changed growth conditions, so that the reduced Ga flow is overcompensated. Borgström et al. reported a comparable effect for doping of InP nanowires using dimethylzinc (DMZn). As the group-III species at the growth front is increased, the doping efficiency is reduced and the enhanced growth rates effectively dilute the dopant incorporation [17].

With these experiments, we have found the relatively small process window (0.04 ≤ IV/III ≤ 0.08) for the successful *n*-type doping of VLS-grown GaAs nanowires with high charge carrier densities using TESn.

**Figure 5:**
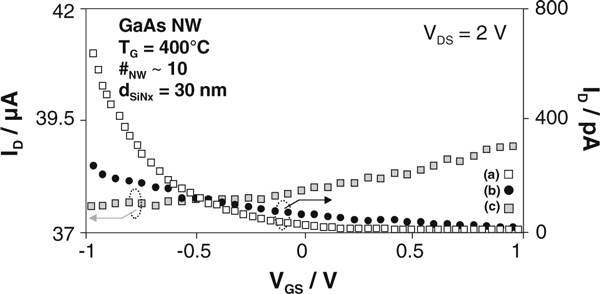
**Transfer characteristics of fabricated multi-channel GaAs nanowire MISFETs with 30 nm SiN_x_ gate dielectric**. The drain-source voltage is 2 V. *a* grown without dopant supply, *b* grown under supply of DitBuSi (IV/III = 0.52), *c* grown under supply of TESn (IV/III = 0.08). Typical *p*-channel behavior is observable for *a*, *b* while *c* proves the *n*-channel behavior of the TESn-doped sample.

Using TESn as dopant precursor implies a *n*-type conductivity of the GaAs nanowires. We fabricated multi-channel MISFET devices with the field-assisted self-assembly (FASA) approach [36], to verify the type of doping. Plotting the drain current I_D_ versus gate-source voltage V_GS_ proves the *n*-channel behavior as the channel conductance increases with positive gate bias (see Figure 5c). Transfer characteristics of the samples grown without dopant supply and grown under supply of DitBuSi show both *p*-channel behavior with currents in the pA range (Figure 5a, b). This can be interpreted easily, since carbon residuals out of the methyl groups may cause *p*-type conductivity. Unfortunately, the gate control of GaAs nanowire MISFET is poor as already reported for nid GaAs nanowires [37] as well as for other materials like GaSb nanowires [38]. This is attributed to a high density of surface states. Effects of such surface/interface states on nanodevices are described and discussed in detail elsewhere [12]. With this measured poor transconductances, we were unable to estimate realistic doping levels.

Finally, this experiment proves the *n*-type doping effect using TESn, which is to our knowledge the first successfully *n*-doped GaAs nanowire grown by VLS in an MOVPE apparatus. An additive proof was given by measuring low and room temperature electroluminescence of axial *pn*-junctions in single GaAs nanowires. More details about this topic will be given in a subsequent study.

## Conclusion

The successful *n*-type doping during the VLS growth of GaAs nanowires is reported using tetraethyltin as doping precursor. DitBuSi shows no doping effect, which is attributed its amphoteric behavior and to the low nanowire growth temperature resulting in a low cracking efficiency. In contrast to *p*-type doping, using diethyl zinc, no influence on the crystal structure was observable, despite relatively high dopant supplies. From the experimental resistance data, we were able to estimate a donor concentration *N*_D_ varying from 7 × 10^17^ cm^-3^ to 2 × 10^18^ cm^-3^. The data spreading is attributed mainly to an axially non-uniform doping profile. Transfer characteristic of multi-channel MISFETs, fabricated from these nanowires, proved that the doping of the nanowire is *n*-type, though the gate control is reduced due to Fermi level pinning and interface states.

The described route for the *n*-type doping of GaAs nanowires is of general interest for all compound semiconductor nanowires and for future nanoscaled devices. It points out fundamental aspects regarding the doping capability using different precursors within MOVPE and should provide the basics to synthesize GaAs nanowire *pn*-junctions, which may act as key element in nanowire optoelectronics.
